# A Comparison of Different Types of Esophageal Reconstructions: A Systematic Review and Network Meta-Analysis

**DOI:** 10.3390/jcm11175025

**Published:** 2022-08-26

**Authors:** Pang-Chieh Hung, Hsuan-Yu Chen, Yu-Kang Tu, Yung-Shuo Kao

**Affiliations:** 1 Division of Thoracic Surgery, Department of Surgery, Shuang Ho Hospital, Taipei Medical University, New Taipei City 235, Taiwan; 2 Graduate Institute of Epidemiology and Preventive Medicine, National Taiwan University, Taipei 106, Taiwan; 3 Department of Radiation Oncology, China Medical University Hospital, Taichung 404, Taiwan

**Keywords:** esophagectomy, reconstruction, meta-analysis

## Abstract

Background: A total esophagectomy with gastric tube reconstruction is the mainstream procedure for esophageal cancer. Colon interposition and free jejunal flap for esophageal reconstruction are the alternative choices when the gastric tube is not available. However, to date, a solution for the high anastomosis leakage rates among these three types of conduits has not been reported. The aim of this network meta-analysis was to investigate the rate of anastomotic leakage (AL) among the three procedures to determine the best esophageal substitute or the future direction for improving the conventional gastric pull-up (GPU). Methods: We searched PubMed, Cochrane, and Embase databases. We included esophageal cancer patients receiving esophagectomy and excluded patients with other cancer. The random effect model was used in this network meta-analysis. The Newcastle–Ottawa Scale (NOS) was used for the quality assessment of studies in the network meta-analysis, and funnel plots were used to evaluate publication bias. The primary outcome is anastomosis leakage; the secondary outcomes are stricture formation, length of hospital stays, and mortality rate. Results: Nine studies involving 1613 patients were included in this network meta-analysis. The trend results indicated the following. Regarding anastomosis leakage, free jejunal flap was the better procedure; regarding stricture formation, colon interposition was the better procedure; regarding mortality rate, free jejunal flap was the better procedure; regarding length of hospital stay, gastric pull-up was the better treatment. Discussion: Overall, if technically accessible, free jejunal flap is a better choice than colon interposition when gastric conduit cannot be used, but further study should be conducted to compare groups with equal supercharged patients. In addition, jejunal flap (JF) cannot replace traditional gastric pull-up (GPU) due to technical complexities, more anastomotic sites, and longer operation times. However, the GPU method with the supercharged procedure would be a possible solution to lower postoperative AL. The limitation of this meta-analysis is that the number of articles included was low; we aim to update the result when new data are available. Funding: None. Registration: N/A.

## 1. Introduction

Esophageal cancer is prevalent in the world. It is ranked as the eighth most common cancer worldwide and has a higher prevalence in Asia and Africa. Recently, the addition of HER2 status, PD-L1 expression, MSI, and overall mutational burden information has improved the overall survival (OS) of esophageal cancer patients, but most patients still present with advanced disease and poor overall survival [1]. The overall five-year survival rate has been estimated to be 15–20% worldwide and it varies widely according to cancer site [2,3]. The mainstream treatment of esophageal cancer is traditionally a combination of surgery and neoadjuvant or adjuvant concurrent chemoradiotherapy. Moreover, a total esophagectomy with radical lymph node dissection is currently the crucial therapy for resectable esophageal cancer and provides a significantly positive impact on OS [4].

**Figure 1:**
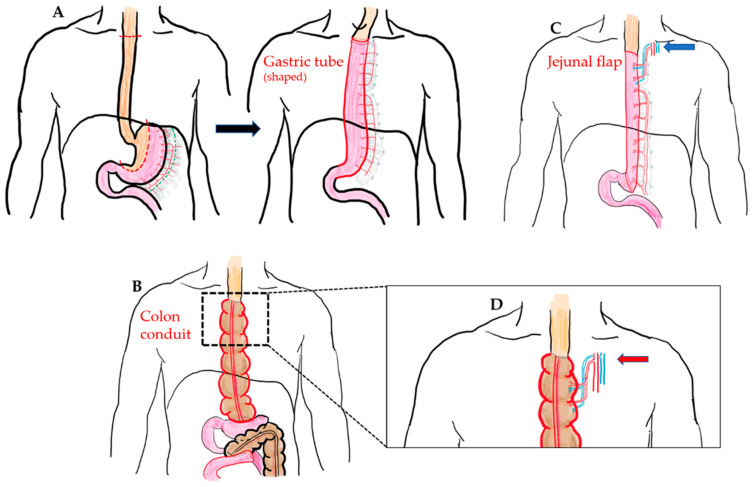
(**A**) McKeown esophagectomy (dotted line: resection margin) and GPU reconstruction (shaped gastric tube pulled-up and anastomosis with proximal end of esophagus at cervical region). (**B**) Illustration of the isoperistaltic CI (use of ascending colon and partial transverse colon as conduit) without supercharged procedure. (**C**) Illustration of the pedicled JF with cervical supercharged procedure (blue arrow). (**D**) Illustration of the additional supercharged procedure at proximal anastomosis in the CI method (red arrow).

Currently, there are several esophageal reconstruction options following a total esophagectomy, including the choice of conduit, route of reconstruction, and the site of anastomosis. The gastric tube is widely accepted as the first choice for an esophageal substitute, which is known as gastric pull-up method (GPU) (Figure 1A). However, in some cases, GPU is not possible or suitable for reconstruction, such as in the case of gastric tumor extension, previous gastric surgery for cervical esophageal or hypopharyngeal cancer, corrosive injury involving the stomach, and failure of a previous gastric conduit [5,6,7]. In these circumstances, to reach the goal of substitute length, sufficient blood supply, and more radical treatment, several types of substitute conduit have been proposed for esophageal reconstruction after total esophagectomy, including colon interposition (CI) (Figure 1B), jejunal flap (JF) (Figure 1C), and skin or anterolateral thigh (ALT) musculocutaneous flaps [8,9]. However, perioperative complications are frequently associated with esophageal reconstruction and remain unsolvable problems among these substitute choices.

Anastomotic leakage (AL) is one of the most disastrous and annoying perioperative complications after an esophagectomy, which can lead to morbidity, hospital mortality, and a negative prognostic impact on long-term survival [10,11]. Although, recently, the mortality rate after AL has decreased due to improvements in surgical techniques and patient selection, the rate of AL is still high, and according to recently reported data is approximately 10–25% [12,13]. AL may further lead to a longer hospital stay, anastomotic strictures, and in-hospital mortality. The mortality rate after AL is approximately 4–10% based on different esophagectomies (transhiatal esophagectomy, McKeown, and Ivor–Lewis) [14]. Several surgical or non-surgical risk factors, such as anastomotic sites (cervical vs. thoracic), intraoperative perfusion assessment, neoadjuvant chemoradiation, higher BMI, diabetes mellitus, and COPD have been investigated thoroughly and have been associated with the development of AL, but these factors have all focused on gastric pull-up (GPU) reconstruction [15,16,17,18]. However, few cohort studies have compared gastric tube reconstruction with CI or JF, which is thought to be the second choice for reconstruction. The aim of this network meta-analysis was to investigate the rate of AL among the three different procedures and to determine the best potential esophageal substitute or the future direction for improving conventional GPU.

## 2. Materials and Methods

The PRISMA-NMA guidelines were used for this study. The following supporting information can be found in the Supplementary Materials.

### 2.1. Literature Search

Two authors (P.-C.H. and H.-Y.C.) independently searched three databases, i.e., PubMed, Cochrane, and Embase. If there was a conflict, it was resolved by consensus. The keywords used for the searches were:

Esophageal cancer;
Esophagectomy;
Gastric pull-up;
Colon interposition;
Jejunal flap;
Anastomotic leakage.

### 2.2. Article Eligibility

The inclusion criteria for selected studies included:

1. Patients with esophageal cancer who underwent open or minimally invasive esophagectomy and reconstruction surgery;
2. Gastric pull-up, free jejunal flap, or colon interposition reconstruction, with or without the supercharged procedure;
3. One of the following data should exist: conduit leaking rate, necrotic conduit rate, or reoperation rate to evaluate whether the conduit functions well;
4. Full text article;
5. Article in English;
6. Published up to 2021 (1991–2021);
7. Adult population;
8. Cohort study, case-control study, or clinical trial.

The exclusion criteria included:

1. Pharyngoesophageal cancer;
2. Hypopharyngeal cancer;
3. Pharyngolaryngectomy;
4. Second operation;
5. Case report and case series.

### 2.3. Data Extraction

Two authors (P.-C.H. and H.-Y.C.) independently extracted data from the literature in the databases. If there was a conflict, it was resolved by consensus.

1. Primary Outcome:
  The primary outcome was anastomosis leakage.
2. Secondary Outcome:
  The secondary outcomes were stricture formation, length of hospital stay, and mortality.

We also extracted the following: the name of the study, the surname of the first author, country, publication year, tumor pathology, tumor location, status of reoperation or not, blood loss, ICU stay, and supercharged or not.

If the treatment included fewer than 10 patients, we excluded that study.

### 2.4. Statistical Analysis

In this network meta-analysis, a generalized linear mixed model was used to analyze the dichotomous data and the restricted maximum likelihood method was performed to estimate the results. For continuous data, a frequentist framework was used to generate the results [19]. 

In the network meta-analysis, when a loop formed in the evidence, we checked whether there was inconsistency between the direct and indirect evidence.

We use the *p*-scores to rank the included treatment.

A sensitivity test was conducted if a study showed a high risk of bias. The statistical analyses were all performed with R language and R studio. R package “Netmeta” was used. *p* values less than 0.05 were statistically insignificant.

### 2.5. Quality Assessment

The Newcastle–Ottawa Scale was used for the quality assessment of the selected studies [20]. Two authors (P.-C.H. and H.-Y.C.) independently scored the included studies and any inconsistencies were resolved by consensus.

### 2.6. Bias Evaluation

Publication biases were evaluated by funnel plots. We performed the Egger’s test when the included article had a score greater than 9.

## 3. Results

**Figure 2:**
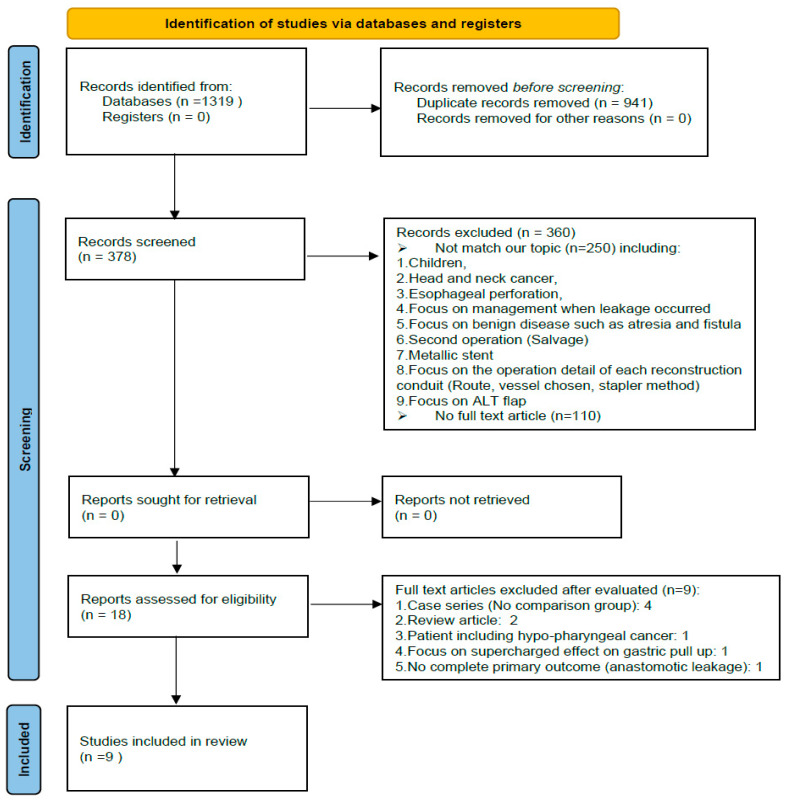
PRISMA flowchart.

Nine studies, involving 1613 patients, were involved in this network meta-analysis. The article selection flowchart is shown in Figure 2; the characteristics of the selected studies are listed in Table 1.

**Table 1:** A summary of included studies.

Author (Ref.)	Study Design	Country, Year	Follow-Up Year	Patient Number	Reconstruction Method				Supercharged	NOS
					Method 1 (*n*)	AL 1 (*n*,%)	Method 2 (*n*)	AL 2 (*n*,%)		
Kolh et al. [21]	Retrospective	Belgium, 2000	1990–1998	130	GPU (92)	6, 6.5%	CI (38)	AL (1.2.6)	No	6
DeMeester et al. [22] *	Questionnaires	USA, 2001	X	201	GPU (116)	11,9.5%	CI (85)	AL (8.9.4)	No	1
Huttl et al. [23]	Questionnaires	Germany, 2002	1999	719	GPU (653)	79, 12.1%	CI (66)	10, 15.1%	No	4
Davis et al. [24]	Prospective	HK, 2003	1982–2000	1001	GPU (959)	37, 3.9%	CI (42)	6, 14.3%	No	8
Briel et al. [11]	Retrospective	USA, 2004	1996–2002	393	GPU (230)	33.14.3%	CI (163)	10, 6.1%	X (no detail)	8
Daiko et al. [25]	Retrospective	Japan, 2007	1982–2002	71	GPU (21)	2.9.5%	JF (50)	2, 4%	X (no detail)	7
Doki et al. [26]	Retrospective	Japan, 2008	1998–2005	49	CI (25)	13.52%	JF (28)	6, 21.4%	Yes (both)	9
Stephens et al. [27]	Questionnaires	USA, 2015	2009–2013	45	GPU (31)	7.22.5%	JF (14)	4, 28.5%	X(no detail)	4
Luan et al. [28]	Retrospective	USA, 2018	2004–2014	100	GPU(85)	7.22.5%	CI (15)	4, 28.5%	Yes (JF)	6
**Author (Ref.)**	**Mean Age**		**Pathology (*n*)**		**Tumor Location**	**Pstage**	**Preoperative Chemoradiotherapy**		**Colon Conduit Choice**	
	**Method 1**	**Method 2**					**Method 1**	**Method 2**		
Kolh et al. [21]	63.4 ± 10.2	52.3 ± 12.8	Adeno: 62 SqCC: 28 Cardia: 33		Upper: 14 Middle: 49 Lower: 33 Cardia: 34	I: 21 II:51 III:52 IV:6	X	X	Light side colon isoperistaltic	
DeMeester et al. [22] *	X	X	X		X	X	X	X	X	
Huttl et al. [23]	X	X	SqCC: 706 Barret: 282		X	X	X	X	Right side colon antiperistaltic	
Davis et al. [24]	62.8 ± 9.3	62 ± 9.7	Adeno: 107 SqCC: 873 other: 21		Cervical: 52 Upper: 64 Middle: 503 Lower: 253 Cardia: 104 Double: 25	0:37 I: 48 II:249 III:553 IV:113	23, 25%	7, 18%	Right side colon antiperistaltic (mostly)	
Briel et al. [11]	X	X	X		X	X	X	X	X	
Daiko et al. [25]	X	X	SqCC: 74		Cervical: 74	I: 6 II:30 III:38	X	X	X	
Doki et al. [26]	63.75 ± 7.2	66.5 ± 7.8	X		X	0:4; I: 7 II:17 III:15 IV:10	9, 35%	8, 35%	Right side colon antiperistaltic	
Stephens et al. [27]	63 ± 10	55 ± 15	Cancer: 39; benign: 6		X	X	X	X	X	
Luan et al. [28]	63.1 ± 13.1	60.2 ± 11.2	X		X	X	30, 35%	6, 40%	X	

* Low study quality; Gastric pull-up = GPU, colon interposition = CI, free jejunal flap = JF, anastomotic leakage = AL, primary outcome = PO, pstage = pathological stage.

### 3.1. Anastomosis Leakage

**Figure 3:**
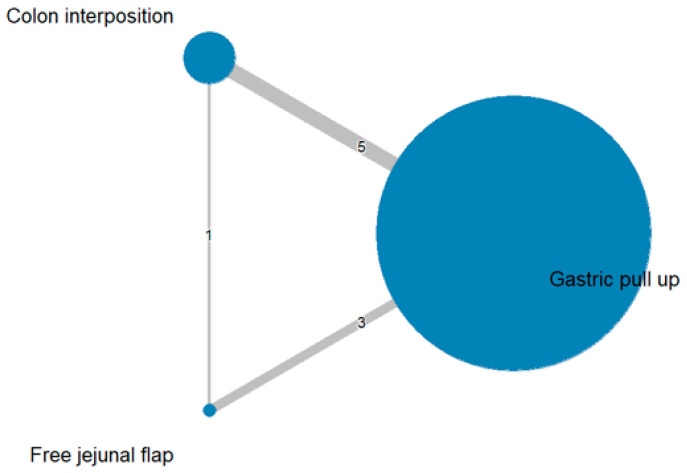
The network plot for anastomosis leakage.

**Figure 4:**
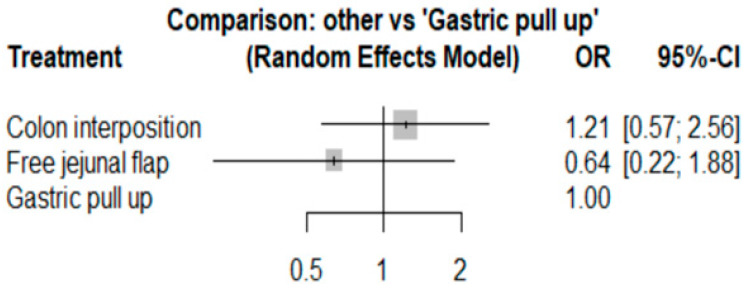
The forest plot for anastomosis leakage.

**Figure 5:**
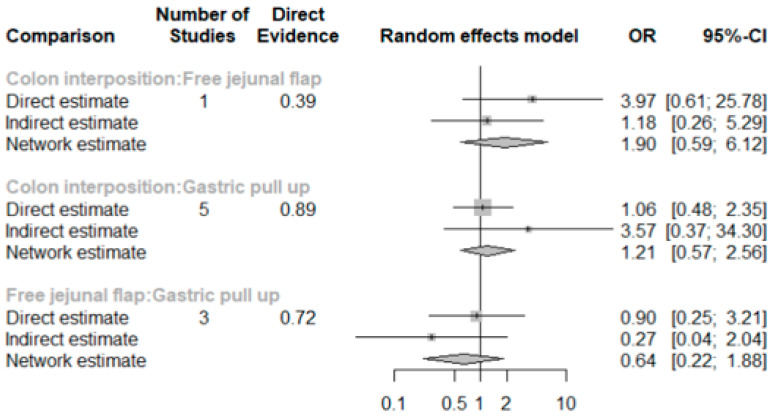
The forest plots for anastomosis leakage (direct and indirect evidence).

The network and forest plots for anastomosis leakage are shown in Figure 3 and Figure 4, respectively. From the forest plot, it is not possible to deduct the best procedure for reducing anastomosis leakage, but the *p*-scores showed that free jejunal flap (0.8255) was better than gastric pull-up (0.4509) and that colon interposition had a high probability of anastomosis leakage (0.2236). Since our network meta-analysis formed a loop at this endpoint, the direct and indirect evidence was analyzed and the results are shown in Figure 5. From the forest plots, it can be observed that there was no inconsistency between the direct and indirect evidence.

### 3.2. Stricture

**Figure 6:**
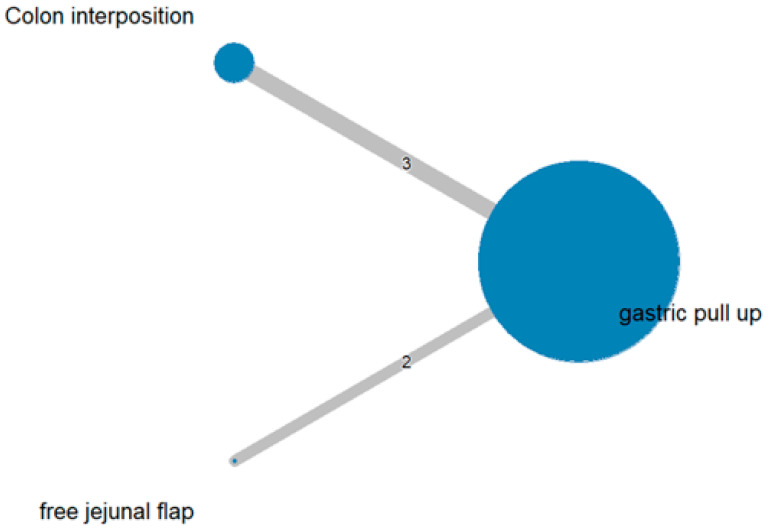
The network plot for stricture formation.

**Figure 7:**
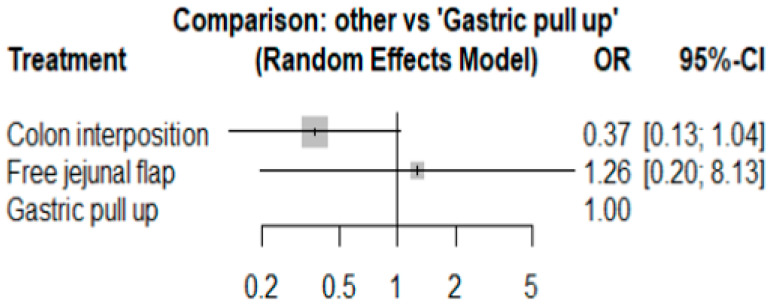
The forest plot of stricture formation.

The network and forest plots for stricture formation are shown in Figure 6 and Figure 7, respectively. From the forest plot, we cannot conclude which procedure is the best for reducing stricture. However, the *p*-scores showed that colon interposition (0.9201) was better than gastric pull-up (0.3131) and that gastric pull-up had a high probability of stricture formation (0.2668).

### 3.3. Mortality Rate

**Figure 8:**
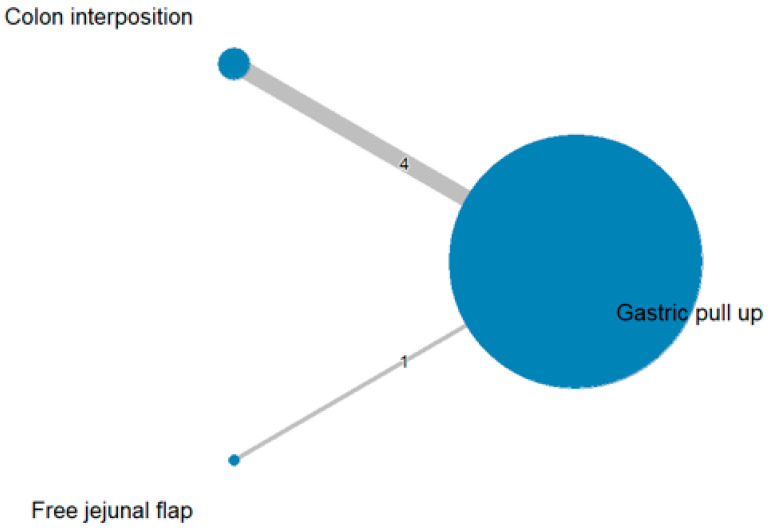
Network plot for mortality rate.

**Figure 9:**
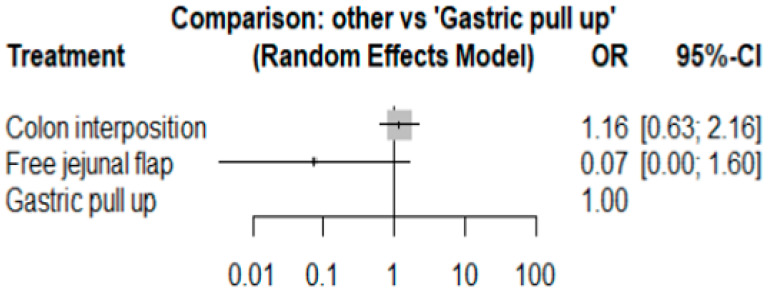
Forest plot for mortality rate.

The network and forest plots for mortality rate are shown in Figure 8 and Figure 9, respectively. From the forest plot, we cannot conclude which procedure is the best for lowering the mortality rate. However, the *p*-scores showed that free jejunal flap (0.9547) was better than colon interposition (0.3665) and that gastric pull-up had a high probability of stricture formation (0.1788). However, only one study compared free jejunal flap with gastric pull-up, and therefore, the results should be used cautiously.

### 3.4. Length of Hospital Stay

**Figure 10:**
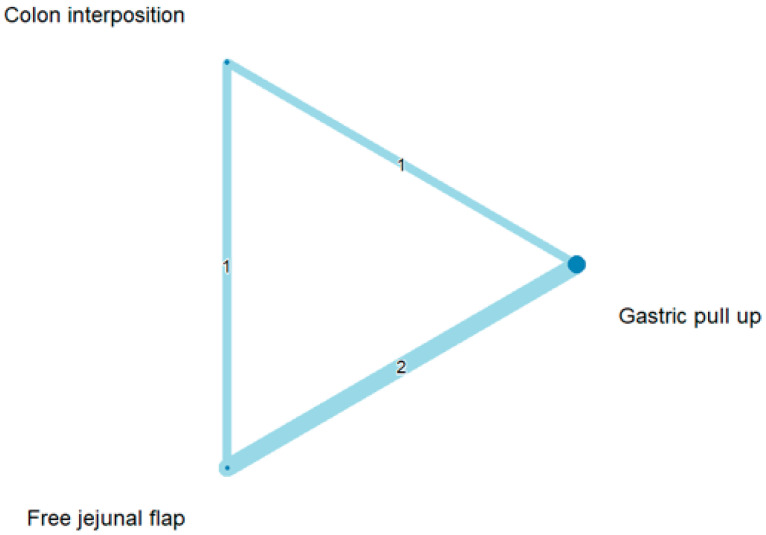
Network plot for hospital stay.

**Figure 11:**
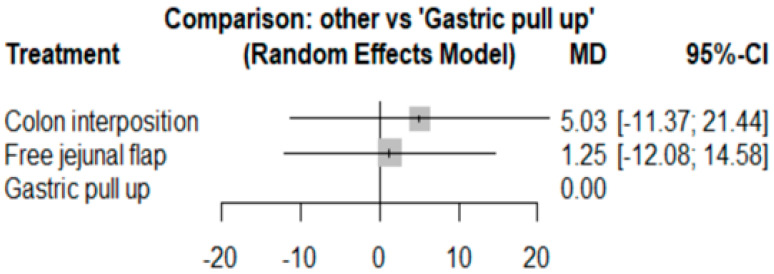
Forest plot for hospital stay.

**Figure 12:**
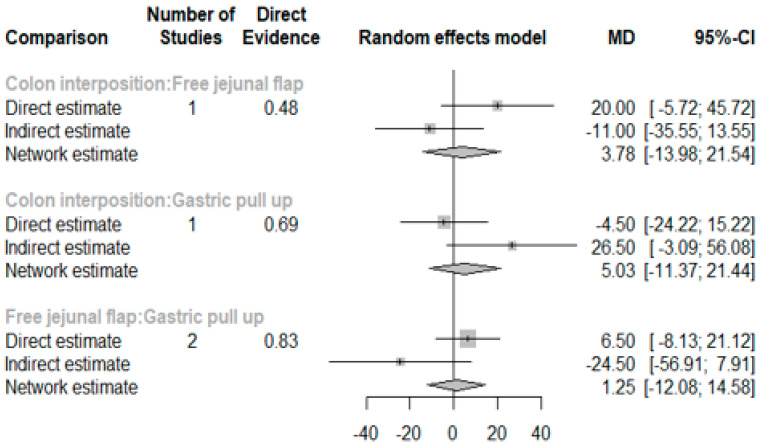
Direct and indirect evidence for hospital stay.

The network and forest plots for length of hospital stay are shown in Figure 10 and Figure 11, respectively. From the forest plot, we cannot conclude which procedure is the best for lowering the length of hospital stay. However, the *p*-scores showed that gastric pull-up (0.6495) was better than free jejunal flap (0.5444) and that colon interposition had a high probability of prolonging the length of hospital stay (0.3060). Since the network plot for this endpoint formed a ring at this endpoint, we further analyzed the direct and indirect evidence, which showed inconsistencies, as shown in Figure 12.

### 3.5. Quality Assessment of Studies

The quality assessment of the studies in the network meta-analysis is shown in Table 2; three studies received a score of 8 or more.

### 3.6. Sensitivity Analysis

**Figure 13:**
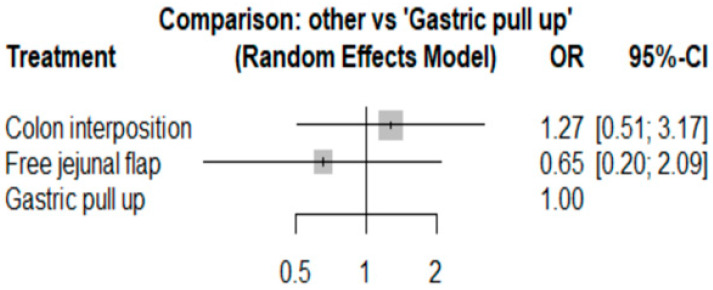
Sensitivity analysis for anastomosis leakage.

Because one article [22] had a low NOS score, we removed it and then conducted a sensitivity analysis, and the results for anastomosis leakage are shown in Figure 13. The *p*-scores were as follows: free jejunal flap, 0.8044; gastric pull-up, 0.4640; and colon interposition, 0.2316. It can be seen that the results in Figure 13 are similar to previous results. Thus, we can conclude that our results are reliable.

### 3.7. Bias Evaluation

**Figure 14:**
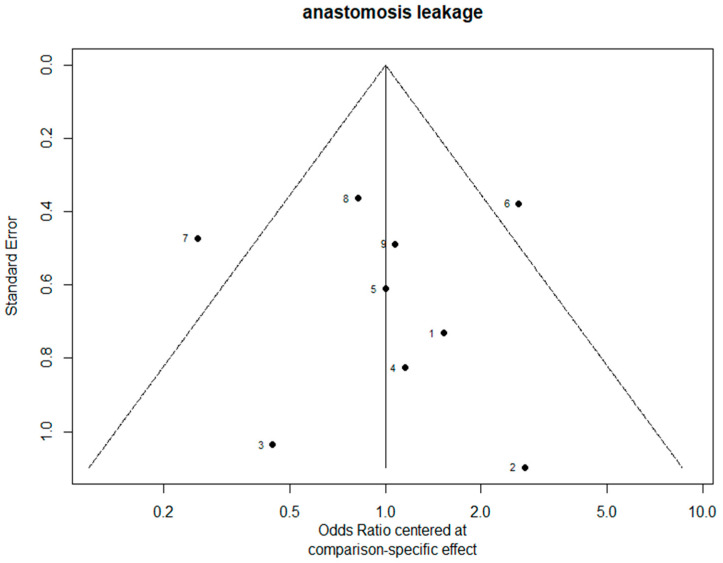

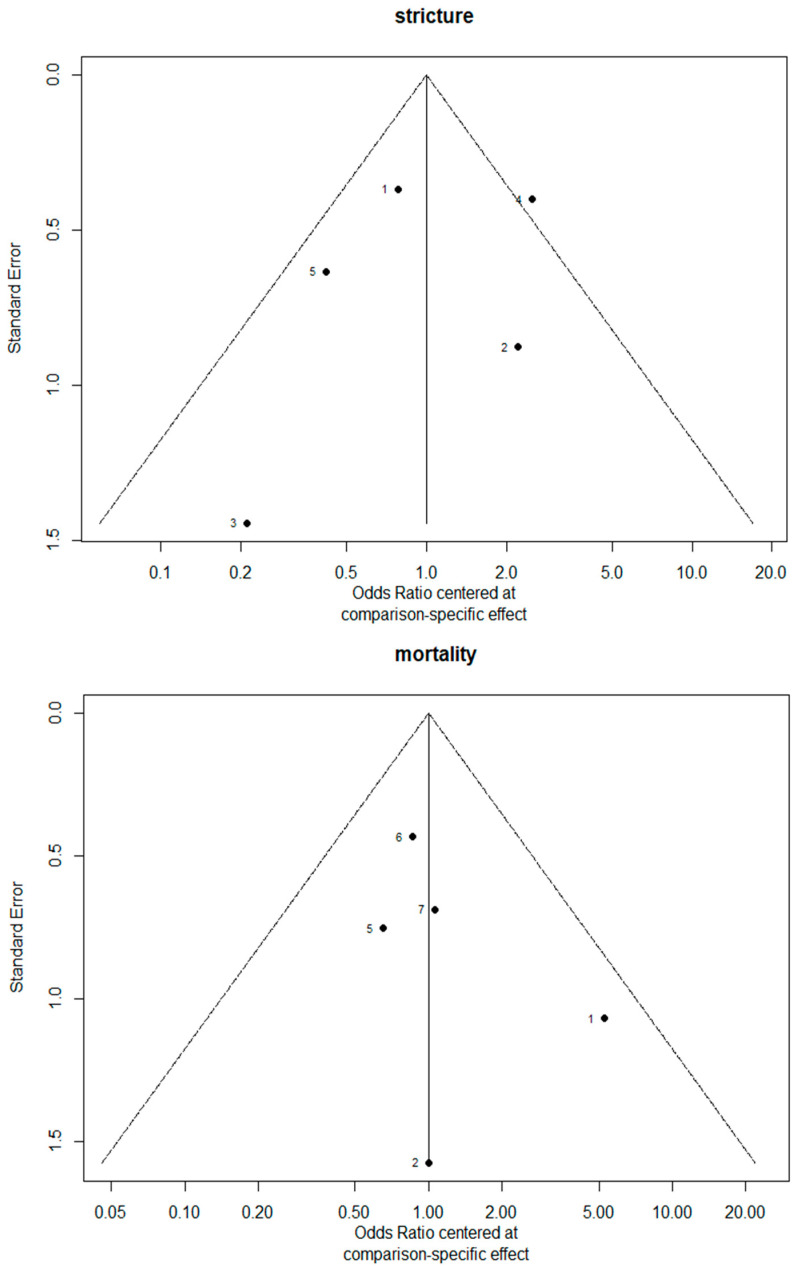

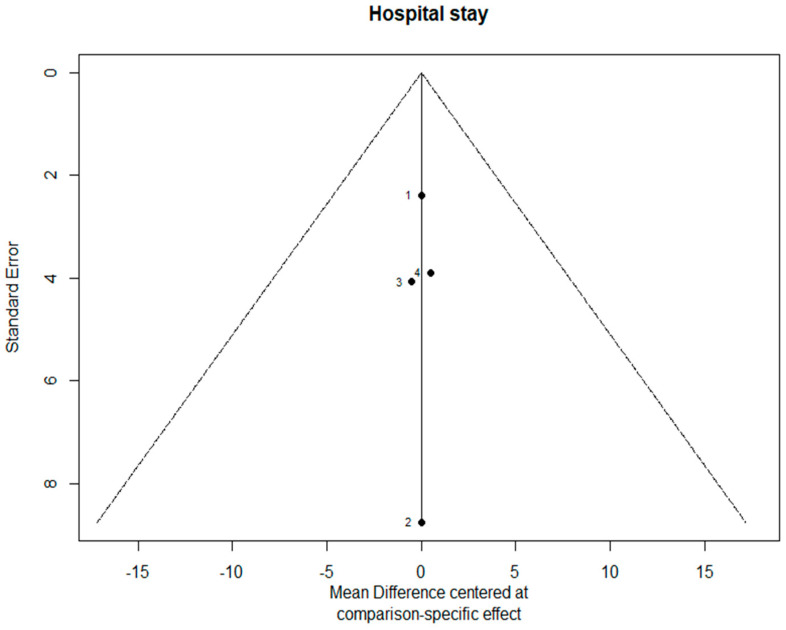
The funnel plots.

The funnel plots are provided in Figure 14. Since there were fewer than 10 studies, we cannot conclude whether or not there was publication bias in this network meta-analysis.

## 4. Discussion

To the best of our knowledge, this study is the first network meta-analysis to compare different reconstruction methods for esophagectomy. Our data revealed no significance among the procedures, but we could see two obvious trends. JF had advantages with respect to AL (*p* = 0.8255), in-hospital mortality (*p* = 0.9547), and length of hospital stay (*p* = 0.8044) as compared with the other reconstruction procedures. Direct and indirect evidence also showed consistent results in post-operative AL (Figure 5). CI was only superior in postoperative anastomotic stricture formation but inferior with respect to other complications. However, the above data should be discussed carefully due to the different patients’ conditions and slightly different indications among the three methods. The indication of GPU is relatively clear for most esophageal cancer patients receiving total esophagectomy. JF and CI were considered if gastric tube was not available or suitable, such as gastric tumor extension, previous gastric surgery for cervical esophageal or hypopharyngeal cancer, corrosive injury involving the stomach, and failure of a previous gastric conduit [5,6,7]. Thus, CI and JF could be compared with each other, but GPU should be discussed separately and it should be considered as indirect evidence to prove whether the method of JF is superior to CI.

During the literature search, the best postoperative outcome was achieved by JF, but this was based on the least clinical data among the three esophageal reconstruction procedures after a total esophagectomy. Theoretically, JF has many advantages over CI, including original rare benign or malignant diseases, the nature of intrinsic peristalsis, and lumen size similar to an esophagus [5]. However, in the real world, JF is often considered to be the third choice among reconstruction methods, due to its complexity, at least three bowel anastomotic sites, two microvascular anastomoses, and the necessity of a hemi-manubriectomy to the reach left internal mammary vessels [5,29]. Moreover, prolonged operation time caused by additional microvascular anastomosis is another obstacle for a surgeon to avoid JF. In two reported case studies, additional operation time ranged from 5 to 20 h, and averaged 7.5 h (not including esophagectomy) [7,29]. Therefore, JF is mainly performed in specific medical centers that have experienced microvascular teams or surgeons. In a case study review of JF, the AL rate ranged from approximately 10% to 32% and the 30-day mortality ranged from 0 to 20% [30].

The Cl procedure is another popular choice for esophageal reconstruction when the stomach is not suitable. In our network meta-analysis, CI had higher leakage rates, longer hospital stays, and higher mortality rates in our meta-analysis. However, these findings may be because the studies included for colon interposition were out of date and most of the colon conduit was performed without the “supercharged” or “super-drained” treatment. The supercharge or super-drained procedure means to perform an additional microvascular anastomosis (artery and vein or vein only) to augment the blood supply of proximal bowel anastomosis (Figure 1D). Formerly, it was used after laryngo-pharyngo-esophagectomy in select patients but, since 2000, it has started to be widely used in CI reconstruction after an esophagectomy [26]. In a review of case studies that compared JF and CI reconstruction, the microvascular supercharged CI procedure was performed in 4 out of 14 case studies with 111/713 (15%) patients and the microvascular supercharged JF procedure was performed in 8 out of 10 case studies with 232/280 (83%) patients [30]. The AL rate ranged approximately from 0% to 46.4% and the 30-day mortality ranged from 0 to 16.3% [21,26,31,32,33]. The additional supercharged procedure could significantly reduce the AL rate and the complications caused by graft ischemia in CI [34]. Demeester et al. also found that in the circumstance of separate origins of the right and left branches of the middle colic artery, using the transverse colon without the supercharged graft was risky [22]. Regarding the incidence of AL, Watanabe et al. found no significant difference between JF and CI; in-hospital mortalities and hospital stays were higher and longer, respectively, in the CI group as compared with the JF group, which were similar to the results of our study [30]. However, the in-hospital mortality rate and hospital stay may not only be influenced by the supercharged procedure but also other factors such as age or resectability [35]. In our study, we could not perform a subgroup meta-analysis to confirm this fact due to loss of patient baseline characteristics among the selected studies, but the different AL outcomes between previous reviews and our study revealed that the blood supply of anastomosis may have a strong influence on the leakage rate. The importance of the supercharged procedure may explain the result of a lower AL rate in the JF group.

GPU is not routinely performed with the supercharged procedure, due to short remnant left gastroepiploic vessels and additional operation time. Traditionally, the blood supply of anastomosis is evaluated intraoperatively by palpitation of conduit temperature or inspection of the conduit serosa. Some investigators have intraoperatively used real-time ICG mapping to evaluate the conduit ischemia condition and chose the best anastomotic position to reduce conduit leakage and necrosis rate, which has been proven by several retrospective and meta-analysis studies [35,36,37,38]. Recently, a prospective comparative study found that the supercharged cervical anastomosis for esophagectomy (SAFE) procedure significantly reduced the postoperative complication rate and hospital stay but increased operation time. Although there was no significant difference in anastomotic leakage rates, all of the patients with the SAFE procedure did not experience leakage as compared with the non-SAFE group of patients (10.9%) [39]. In our study, JF seemed to be a better choice than GPU after an esophagectomy, but the technical complexity, longer operation time, and lack of experienced microvascular surgeons were the main obstructions associated with performing the supercharged procedure. This suggests that different reconstruction methods may not be the cure for post-esophagectomy AL; however, emphasizing the supercharged procedure itself could greatly lower the AL rate.

Our literature search showed that there is a lack of retrospective and prospective comparison studies that have focused on reconstruction methods. Although there are many small sized case studies of JF and Cl, they could not be compared due to diverse baseline patient characteristics, different surgeons, regions, races, and even the details of the reconstruction method. There is still no consensus about the best surgical techniques for JF and CI, such as the choice of supercharged vessel, retrograde or anterograde CI, and reconstruction route (substernal, posterior mediastinum and subcutaneous).

This network meta-analysis has many limitations. First, three of nine included studies did not have a patient’s detailed pathological report which could not identify the purpose of esophagectomy. Second, six of nine studies did not reveal the neoadjuvant chemoradiation status and five of nine studies did not reveal the clinical or pathological cancer stage of the patients which were thought to be the main risk factor of postoperative AL (Table 1B). Third, only four studies were included in the analysis of hospital stay length and there is inconsistency between the direct evidence and indirect evidence. Future meta-analysis studies should collect more comparative literature to further analyze the mentioned factors above.

## 5. Conclusions

Overall, if technically accessible, JF is a better choice than Cl when gastric conduit cannot be used, but further study should be conducted to compare groups with equal supercharged patients. In addition, JF cannot replace traditional GPU due to technical complexities, more anastomotic sites, and longer operation times. However, the GPU method with the supercharge procedure could be the possible solution to lower postoperative AL.

## Figures and Tables

**Table 2:** Newcastle–Ottawa scale.

Author (Ref.)	Selection of Cohorts				Comparability of Cohorts	Outcome			Overall
	Representativeness of the Exposed Cohort	Selection of the Non-Exposed Cohort	Ascertainment of Exposure	Demonstration That Outcome of Interest Was Not Present at Start of Study		Assessment of Outcome	Was Follow-Up Long Enough for Outcomes to Occur	Adequacy of Follow-Up of Cohorts	
Kolh et al. [24]	★	★	★	★	★		★		6
DeMeester et al. [22] *		★							1
Huttl et al. [23]	★	★		★	★				4
Davis et al. [24]	★	★	★	★	★★	★	★		8
Briel et al. [11]	★	★	★	★	★	★	★	★	8
Daiko et al. [25]	★	★	★	★		★	★	★	7
Doki et al. [26]	★	★	★	★	★★	★	★	★	9
Stephens et al. [27]	★	★			★		★		4
Luan et al. [28]	★	★	★			★	★	★	6

★: appropriate study design; ★★: one for the most important factor; the other for another factor. * low study quality.

## Data Availability

The database supporting the conclusion of this article is included within the article and Supplementary Materials.

## Supplementary Materials

The following supporting information can be downloaded at: https://www.mdpi.com/article/10.3390/jcm11175025/s1, File S1: PRISMA 2020 for abstracts checklist; File S2: PRISMA 2020 checklist. See [40].
